# Community health worker-led household screening and management of neonatal hyperbilirubinemia in rural Bangladesh:
 a cluster randomized control trial protocol

**DOI:** 10.12688/gatesopenres.14033.1

**Published:** 2023-04-21

**Authors:** Eric M. Foote, Farjana Jahan, Mahbubur Rahman, Sarker Masood Parvez, Tasnim Ahmed, Rezaul Hasan, Farzana Yeasmin, Shams El Arifeen, Sk Masum Billah, Md. Mahbubul Hoque, Mohammod Shahidullah, Muhammad Shariful Islam, Vinod K Bhutani, Gary L Darmstadt

**Affiliations:** 1 Prematurity Research Center, Department of Pediatrics, Stanford University School of Medicine, Stanford, CA, USA; 2 Environmental Intervention Unit, International Centre for Diarrheal Disease Research, Bangladesh, India; 3 Children's Health and Environment Program, Child Health Research Centre, The University of Queensland, QLD, Australia; 4 Maternal and Child Health Division, International Centre for Diarrheal Disease Research, Bangladesh, India; 5 Faculty of Medicine and Health, Sydney School of Public Health, The University of Sydney, Sydney, Australia; 6 Department of Neonatology, Bangladesh Shishu Hospital & Institute Dhaka, Bangladesh, India; 7 Bangabandhu Sheikh Mujib Medical University, Bangladesh, India; 8 National Newborn Health Program (NNHP) and Integrated Management of Childhood Illness (IMCI), Directorate General of Health Services, Bangladesh, India

**Keywords:** neonatal hyperbilirubinemia, community health workers, low to middle income country, global health

## Abstract

**Background**

Extreme hyperbilirubinemia leading to neurologic disability and death is disproportionately high in low to middle income countries (LMIC) such as Bangladesh, and is largely preventable through timely treatment. Of the estimated 50% of newborns born at home in LMICs born at home, few receive screening or treatment for hyperbilirubinemia, leading to 6 million newborns per year who need phototherapy treatment for hyperbilirubinemia but are untreated. Household screening and treatment for neonatal hyperbilirubinemia with phototherapy administered by a trained community health worker (CHW) may increase indicated treatment for neonatal hyperbilirubinemia in comparison to the existing care system in Bangladesh.

**Methods**

530 Bangladeshi women in their 2 ^nd^ or 3 ^rd^ trimester of pregnancy from the rural community of Sakhipur, Bangladesh will be recruited for a cluster randomized trial and randomized to the intervention arm — home screening and treatment for neonatal hyperbilirubinemia — or the comparison arm to receive usual care. In the intervention arm, CHWs will provide mothers with two prenatal visits, visit newborns by 2 days of age and then daily for 3 days to measure transcutaneous bilirubin (TcB) and monitor clinical danger signs. Newborns without danger signs but with a TcB above the treatment threshold <15 mg/dL will be treated with light-emitting diode (LED) phototherapy at home. Newborns with danger signs or TcB >15 mg/dL will be referred to a hospital for treatment. Treatment rates for neonatal hyperbilirubinemia in each arm will be compared.

**Conclusion**

This study will evaluate the effectiveness of CHW-led home phototherapy to increase neonatal hyperbilirubinemia treatment rates in rural Bangladesh. LMICs are expanding access to postnatal care by using CHWs, and our work will give CHWs a curative treatment option for neonatal hyperbilirubinemia. Similar projects in other LMICs can be pursued to dramatically extend healthcare access to vulnerable newborns with hyperbilirubinemia.

**Trial Registration**: The trial was registered at clinicaltrials.gov on May 1, 2019 (NCT03933423). https://clinicaltrials.gov/ct2/show/NCT03933423?term=NCT03933423&draw=2&rank=1

## Introduction

### Background

Approximately 18% of all infants, including approximately 14 million infants per year in low to middle income countries (LMIC), are at risk of neonatal hyperbilirubinemia progressing to extreme hyperbilirubinemia and brain damage ^1^. Of the 481,000 yearly cases of extreme hyperbilirubinemia, 80% occur in LMICs ^1^. The highest burdens of extreme hyperbilirubinemia are in sub-Saharan Africa and South Asia, where the mortality rate of extreme hyperbilirubinemia and rhesus disease is about 100 times higher and rates of neurologic disability are about eight times higher than rates in high income countries ^1^.

Too often, newborns in LMIC present at hospitals for treatment too late, having already developed brain damage from extreme hyperbilirubinemia when phototherapy is no longer effective ^2^. It is estimated that 6 million newborns in LMICs need treatment yearly yet do not receive it ^3^. There are a cascading series of delays in diagnosis and treatment that occur resulting in preventable disability and deaths of this time dependent condition ^4^. In many LMICs, more than half of newborns are born at home, where postnatal visits and physical exam screenings or laboratory screening for neonatal hyperbilirubinemia are rarely performed ^5^. The World Health Organization (WHO) in the Integrated Management of Childhood Illness (IMCI) guidelines, recommend neonates in LMICs receive home or clinic-based physical exam screenings followed by a referral to a hospital if there is concern for hyperbilirubinemia ^6, 7^. Physical exam based screening by physicians prior to hospital discharge has been shown to be less effective in reducing cases of severe hyperbilirubinemia than transcutaneous bilirubin (TcB) screening ^8^. In LMICs, facility-based vaginal births are often discharged without bilirubin screening after less than 24 hours leading to missed cases of severe hyperbilirubinemia ^8^. Parents often only realize that their child is sick when their newborn develops symptoms from brain damage.

Even if newborns are diagnosed in a timely fashion, hospital-based treatment for newborns in LMICs is difficult to access and expensive for families. In a study on neonatal sepsis in rural Bangladesh, only one-third of newborns that were referred to the hospital from home for physical exam findings concerning for sepsis went to the hospital ^9^. The costs of bringing the newborn to the hospital and obtaining care were found to be major barriers ^9, 10^. However, families were willing to have their newborns treated at home. Approximately 65% of parents who refused referral for neonatal sepsis evaluation in hospitals consented to home care by CHWs with intramuscular injection with antibiotics, increasing access to care and reducing neonatal mortality by 34% ^9^. By providing household treatment for neonatal hyperbilirubinemia, essential care can be expanded to families that would otherwise not have access to treatment potentially reducing disability and death from neonatal hyperbilirubinemia. Families will not have to travel to hospitals, wages will not be lost from taking days off work, the cost of nurses and physicians will be replaced with less expensive and easier to train CHWs, and hospital bed costs will be eliminated. The risk of newborn exposure to hospital borne infections would also be eliminated. Home screening and treatment for neonatal hyperbilirubinemia has the promise to increase access to treatment and reduce cases of extreme hyperbilirubinemia ^11^.

### Study aims and hypothesis

We hypothesize that CHW-led household screening and treatment for neonatal hyperbilirubinemia will increase the rate of indicated treatment for neonatal hyperbilirubinemia when compared to current practices in rural Bangladesh.

***Aim 1 (Prevention)***

Develop and conduct two maternal prenatal educational sessions supervised by CHWs in intervention households to encourage breastfeeding by one hour of life, exclusive breastfeeding through four weeks of age, and help mothers develop a plan for how to get to the nearest hospital with newborn services if their newborn is sick.

We will demonstrate:

- An absolute increase of at least 20% in breastfeeding rates by one hour of age and exclusive breastfeeding rates through four weeks of age in intervention families

***Aim 2 (Screening)***

During appropriately timed home visits, CHWs will screen infants for neonatal hyperbilirubinemia.

We will demonstrate:

- At least 80% of newborns born vaginally in intervention households are screened for neonatal hyperbilirubinemia by two days of age.

***Aim 3 (Treatment)***

Assess the impact of CHW-administered home screening and neonatal hyperbilirubinemia treatment on treatment rates in comparison to the current postnatal care system in Bangladesh.

- The percentage of newborns that receive treatment for neonatal hyperbilirubinemia in the intervention and comparison clusters will be compared.
- We will evaluate the safety of CHW-administered LED phototherapy. We will measure the number of referrals for danger signs after starting home phototherapy, the need for exchange transfusion after starting home phototherapy, the development of extreme hyperbilirubinemia after starting home phototherapy (total serum bilirubin (TSB) ≥ 25 mg/dL, the development of severe hyperbilirubinemia (TSB ≥ 20 mg/dL) after starting home phototherapy, sepsis diagnosed after starting home phototherapy, and newborns diagnosed with hypoglycemia after starting home phototherapy.
- The acceptability of home and hospital hyperbilirubinemia screening and phototherapy treatment will be assessed qualitatively.

### Trial design

We will conduct a prospective cluster randomized control trial to evaluate the impact of CHW-led home screening and treatment for neonatal hyperbilirubinemia on the percentage of newborns treated for hyperbilirubinemia.

In the intervention arm, CHWs will provide home hyperbilirubinemia screening and home treatment with phototherapy or referral for hospital treatment. In the comparison arm, newborns will receive treatment based on the current postnatal care system in rural Bangladesh, where newborns are referred for evaluation for hyperbilirubinemia based on parental concern and physical exam findings during postnatal visits.

## Methods

### Study design

***Study setting***

This study will enroll pregnant mothers in Sakhipur, Bangladesh, which is a rural agrarian community with a population of 300,000. It is 429 square kilometers in area, includes 132 villages, and is a part of the Tangail District. There are approximately 7,000 births in this community per year and approximately half of births occur at home ^12^. There is a government hospital with 50 beds that provides caesarean section, vaginal birthing services, and emergency services ^12^. Poverty, crowding, unstable housing, food insecurity, and poor hygiene and sanitation are common throughout the region.

***Study population***

About 98% of pregnant women in Sakhipur receive at least one antenatal care visit from a trained medical provider, and 66% of women receive four antenatal care visits ^12^. Approximately half of the mothers deliver at home ^12^. Approximately 60% of newborns are seen by a health care provider within the first 48 hours of life ^12^. If neonatal hyperbilirubinemia is diagnosed and phototherapy is needed, newborns are referred for phototherapy treatment to the nearest hospital, which is more than two hours away.

***Sample size***

Approximately 18% of infants need treatment for neonatal jaundice ^1^. We estimate that of the neonates born in facilities, half of those in need of treatment actually receive the indicated treatment and for home births, none receive the indicated treatment ^1^. The indicated treatment rate in the comparison arm will be 25%, or 4.5% of infants. In the intervention arm, we expect that we can provide home phototherapy in 60% of cases of neonatal hyperbilirubinemia that need treatment. Of the other 40% of infants that we identified and referred to the hospital for treatment, we estimate that 33% will receive treatment ^9^. The total indicated treatment rate is estimated at 80%, or 14.4% of infants. With a type 1 error rate of 5% and type 2 error rate of 20%, and a design effect of 1.6 due to cluster sampling and a loss to follow-up of 5%, the sample size is 262 in each arm. We aim to enroll 530 pregnant mothers from the Sakhipur sub-district.

### Participant recruitment

The trial received oversight from the steering committee composed of eight investigators from icddr,b, three investigators from Stanford University, one investigator from Dhaka Shishu Hospital, and one investigator from Bangabandhu Sheikh Mujib Medical University.

Trained fieldworkers from icddr,b will travel to the eligible communities and ask community leaders for permission to conduct research within their community. If the community leaders agree, then the team will proceed with recruitment. The prospect of participation in the study will be discussed with adults in the communities, including the pregnant mothers. The scientific field team will go house-to-house to list all the pregnant women on the basis of documentation of their pregnancy ( *i.e.* Antenatal care (ANC) card and/or prescription from the selected unions of a sub district) to enroll them as study participants if they meet the eligibility criteria. If any pregnant mother does not have an ANC card, but is willing to participate in the study, she will be sent to local health facilities to register for an ANC card. Before recruitment, we will reach out to local government workers to identify pregnant women in 2 ^nd^ or 3 ^rd^ trimester. They maintain a register of all newlyweds and update new pregnancies to encourage ANC, safe delivery and postnatal care services. In addition, the trained fieldworkers will travel to the eligible communities to create an enabling environment to conduct the research in the community. Written informed consent will be obtained from interested participants prior to beginning the study.

***Inclusion criteria***

Study participants will be pregnant women 18 years of age or older in their second or third trimester of pregnancy and who consent to enroll as study participants.

***Exclusion criteria***

Study participants will be excluded if there is a multiple gestation pregnancy, future plans to leave the area within the next 12 months (if a mother is planning to give birth at her natal home and then return, she will not be a candidate for enrollment), if maternal danger signs are present, if there is a history of severe mental health condition, defined as any mental condition either medically diagnosed or reported by family to affect activities of daily living.

There are no inclusion or exclusion criteria for newborns born to study participants. All newborns born to enrolled mothers will be enrolled. A timeline of the study period is described in Table 1.

**Table 1.** Study timeline.

	Study period							
	Enrolment	Allocation	Post-allocation					Close- out
Timepoint**	Month (M) 1–3	M 1–3	M 4–5	M6–7	M8–9	M 10–11	M 12–13	M 14–15
**Enrolment:**								
**Eligibility screen**	X							
**Informed consent**	X							
**Baseline survey, blood grouping of parents**	X							
**Allocation**	X	X						
**INTERVENTIONS:**								
**[Intervention 1-Prenatal educational sessions]**			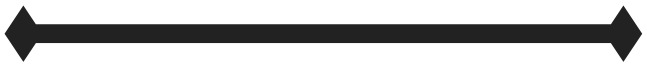					
**[Intervention 2-** Transcutaneous bilimeter screening for **neonatal hyperbilirubinemia ]**				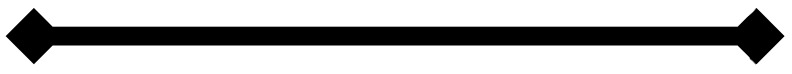				
**Intervention 3: Home treatment or ** **hospital referral for treatment for neonatal ** **hyperbilirubinemia**				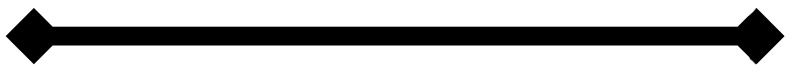				
**ASSESSMENTS:**								
**[Socio-demographic variables, obstetrical ** **history, antenatal care history, medical history,** ** breastfeeding, neonatal hyperbilirubinemia** ** knowledge, attitude and practice]**	X	X						
**[breastfeeding within 1 hour of child birth, ** **hyperbilirubinemia screening within 48 hours of** ** birth, treatment for neonatal hyperbilirubinemia]**				X	X	X	X	X
**Changes in knowledge, attitude, practice for** ** breastfeeding and neonatal hyperbilirubinemia**				X	X	X	X	X

### Cluster randomization and intervention allocation

**Figure 1.**
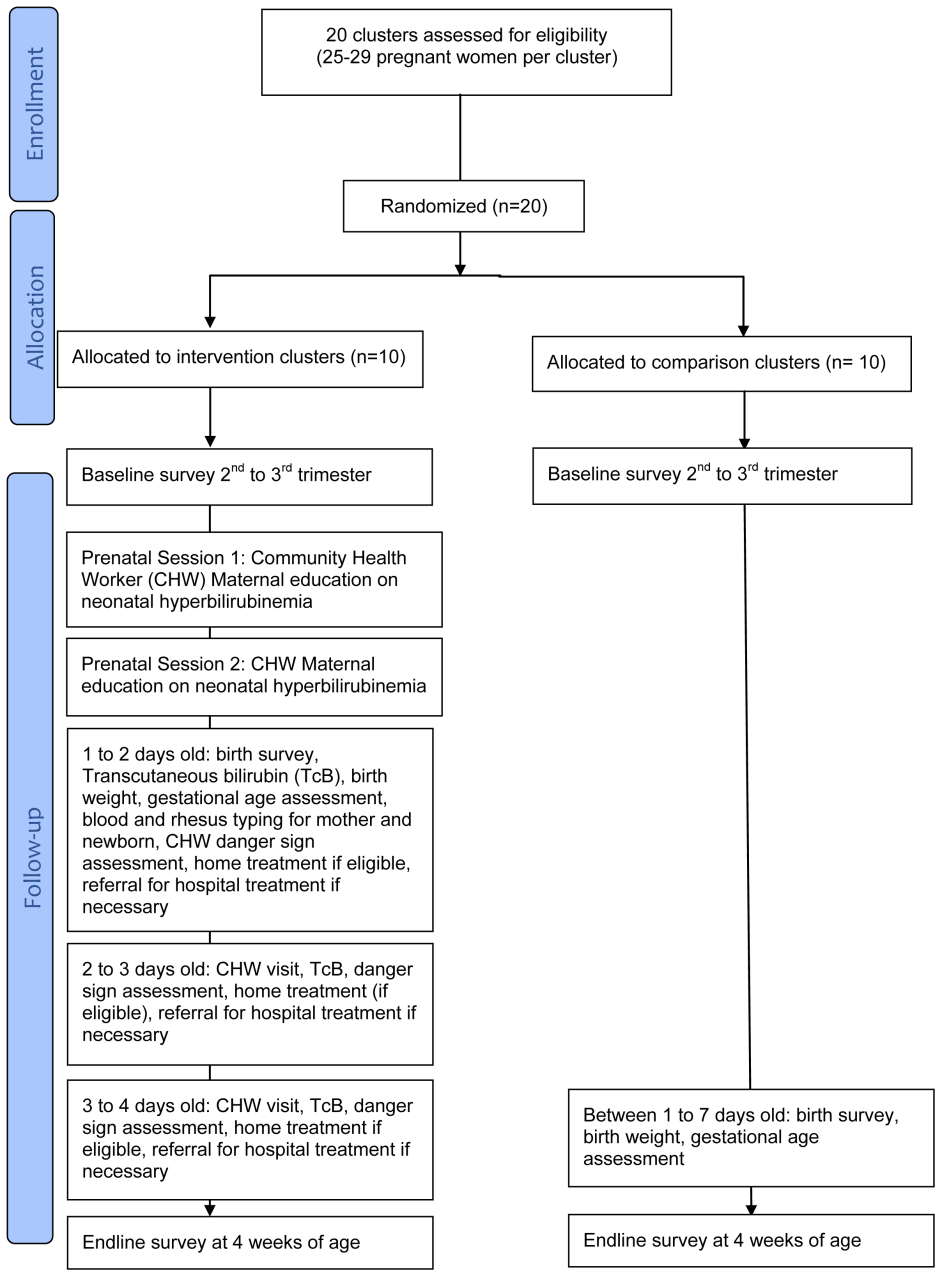
Randomization scheme: CONSORT flow diagram.

Amongst eligible participants, we will form clusters of 25–29 expectant mothers who live close enough based on the geographical proximity ( Figure 1). Households that do not form a cluster according to geographical proximity will not be included in the cluster. Finally, a statistician not involved in the study will randomly assign 20 clusters using a random number generator. Clusters will be divided equally across intervention and control arms.

Given the nature of treatment in the intervention group, masking of participants and caretakers will not be feasible in the intervention group. CHWs will know if they are delivering the intervention. However, care will be taken to ensure that the outcome assessors are not aware of the interventions allocated to specific clusters. Individuals involved in delivering the intervention and the evaluation will have minimal contact to minimize bias.

## Community health workers recruitment and training

CHWs will be recruited through local advertisements and supervised by the Bangladesh-based scientific team. The eligibility criteria to become CHWs include female gender (due to cultural sensitivities with providing breastfeeding support), at least 20 years of age, completion of secondary school, and a passing score on a written test. 12 CHWs who meet the requirements for initial CHW enrollment will be invited for a three-day long training conducted by icddr,b staff and physicians from Dhaka Shishu Hospital. The training model is adopted from the “Validation of community health workers’ assessment of neonatal illness in rural Bangladesh” by Darmstadt et al. This will include training on prenatal and postnatal care, antenatal counseling on preparedness for birth and newborn care, management of neonate at birth, essential newborn care, routine neonatal assessment, newborn danger sign classification, management of illness, and breastfeeding assessment. The training will be conducted at the local government hospital where the CHWs will gain familiarity with the referral center.

During the first phase on training, CHWs will be assessed through a pre- and post- training knowledge evaluation. Based on the performance, 10 CHWs will be selected for recruitment and second phase training. The CHWs will then have two days of theoretical and practical training on weight measurement, TcB measurement, temperature measurement, lactation assessment, and newborn danger sign assessment. CHWs will be trained to make newborn danger sign and lactation assessments in the hospital under the supervision of physicians and receive feedback on their assessment of five newborns in the hospital. The CHWs will also have three days of training on phototherapy device use in the hospital. Each CHW will observe the management of care of three newborns in the hospital under the guidance of a neonatologist. Then, the CHWs will carry out assessments in the field for three days under observation from the research physician.

We will also conduct a training session with the staff nurses and medical officers of the referral hospital to orient them to the study intervention and ensure proper management of referred newborns. Field staff along with the training team will review performance (knowledge, skill, and attitude) of CHW’s every two weeks and provide advice and trainings to maintain skills.

Each CHW will manage the care of approximately twenty newborns per month over the course of the study. On average each CHW will perform three home visits per day. Study households will be within one-hour travel time for the CHWs.

### Interventions

***Intervention arms***

**Intervention 1: Breastfeeding and neonatal hyperbilirubinemia module development**

We will develop a CHW-led prenatal education module to encourage early and frequent breastfeeding by one hour of life, exclusive breastfeeding through one year of age, and to educate mothers on the neonatal and maternal danger signs as well as the dangers of severe neonatal hyperbilirubinemia. The module will help mothers develop a plan to bring their newborn to an appropriate health facility if necessary, after birth. We will adapt the guideline from Bangladesh Ministry of Health Comprehensive Newborn Care package ^13^. We will share the guideline with our key stakeholders to incorporate their recommendations to make the modules more feasible for CHWs. CHWs will visit enrolled pregnant mothers and conduct two prenatal education sessions which will increase awareness among mothers and other household members about exclusive breast feeding and risk of neonatal hyperbilirubinemia. The sessions will include education on the importance of attending recommended antenatal and postnatal newborn and maternal care visits, delivery preparation, ante partum, partum and post-partum danger signs, neonatal danger signs, exclusive breastfeeding, and neonatal hyperbilirubinemia (including signs, symptoms, screening *via* transcutaneous bilimeter and possible need for treatment in the hospital or at home with phototherapy). Newborns in the intervention arm are not restricted from receiving neonatal hyperbilirubinemia care through the existing postnatal care system.

**Intervention 2: Blood and rhesus typing, noninvasive TcB screening newborns for neonatal hyperbilirubinemia and evaluation of danger signs**

The mother’s blood and rhesus type will be determined at the birth survey visit if it was not already determined during antenatal visits. Newborn blood grouping and rhesus typing will be determined at the birth survey visit. There will be three drops of blood collected using a lancet needle. For newborns the specimen will be taken from the heel and for mothers it will be taken from the finger. The slide test method will use anti-monoclonal blood type antibodies for each blood type and anti-D monoclonal antibodies. We define rhesus incompatibility to be when the mother is rhesus negative and the newborn is rhesus positive, and we define blood type incompatibility to be when the mother is blood group O and the newborn does not have blood group O. If rhesus incompatibility is present, we will refer the mother for rho(D) immune globulin treatment at a local health facility if she has not already received treatment.

**Figure 2.**
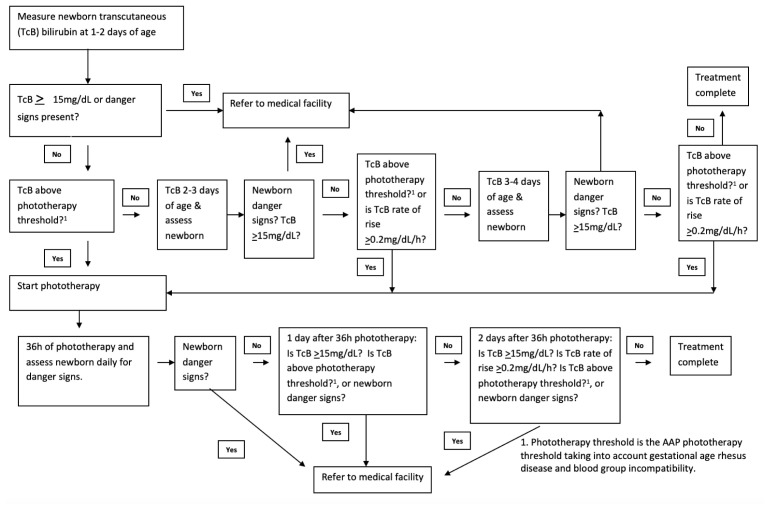
Neonatal hyperbilirubinemia management algorithm.

**Figure 3.**
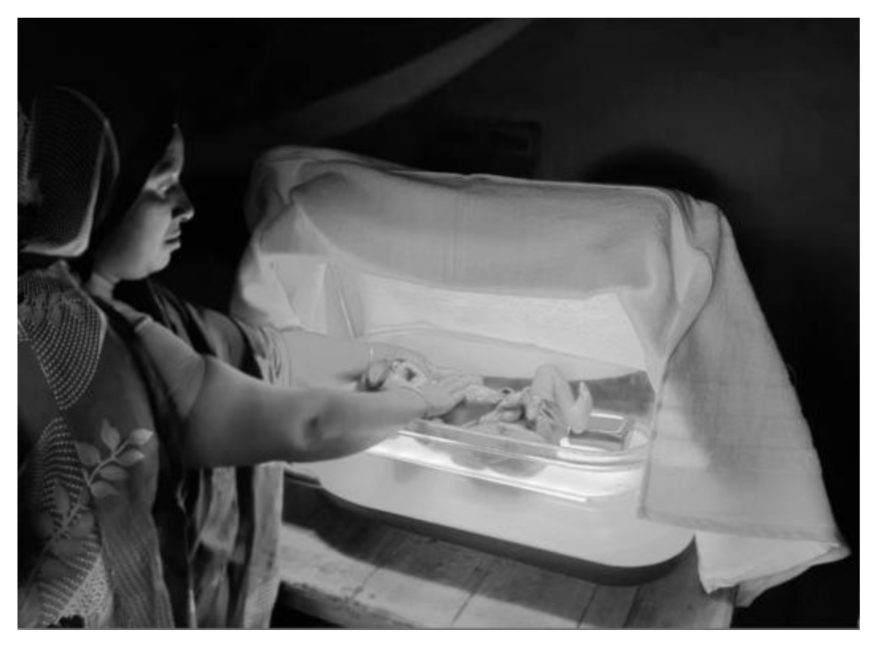
Phototherapy device in use with white blanket covering (Firefly) (Source: icddr,b).

We will follow the neonatal hyperbilirubinemia management algorithm in Figure 2 that we developed following American Academy of Pediatrics (AAP) guidelines, using intensive >30 μW/cm2/nm irradiance, clinically approved, LED phototherapy (Firefly, Figure 3) ^14^. CHWs will be supported by an electronic tablet that guides them through the clinical management algorithm ( Figure 2) step by step. 

Total bilirubin will be measured using a transcutaneous bilimeter (Draeger JM-105). Transcutaneous bilimeters tend to overestimate the serum bilirubin concentration at TcB levels less than 15 mg/dL by 0.84 mg/dL ^15^. This provides a safety margin as transcutaneous bilimeters tend to measure higher than the serum bilirubin. At levels of bilirubin greater than 15 mg/dL, transcutaneous bilimeters do not approximate the serum concentration as well, which is why we will refer all newborns with a transcutaneous bilirubin greater than 15 mg/dL to the hospital to obtain a serum bilirubin and evaluation by a medical provider ^15, 16^.

The assigned CHW will visit each newborn born vaginally by two days of age and newborns born *via* caesarean section the day after hospital discharge then daily for three consecutive days to assess for hyperbilirubinemia and evaluate breastfeeding and institute phototherapy if indicated. During CHW visits, if any danger signs are observed, CHWs will refer newborns to an appropriate medical facility. The phototherapy treatment level is the AAP phototherapy threshold adjusted for gestational age, and presence of rhesus or blood type incompatibility or if the bilirubin is rising greater than or equal to 0.2 mg/dL/hour on consecutive visits. Newborns eligible for home treatment will be treated with CHW-led LED phototherapy.

The following neonatal and maternal danger signs were adopted from the National Neonatal Health Strategy 2009 and research done in Bangladesh to validate neonatal danger signs to predict need for hospital-based care ^17– 20^. If any of the following neonatal danger signs are present on CHW assessment, the newborn will be referred to nearest appropriate medical facility for treatment.

1. Poor feeding
2. History of convulsions
3. Tachypnea (>60 breath/min on two consecutive readings)
4. Severe chest in-drawing
5. Hypothermia (less than 35.5°C or 95.9°F)
6. Fever (more than 37.5 °C or 99.5°F)
7. Movement only when stimulated or no movement at all
8. Umbilical redness extending to abdominal skin
9. No void within 24 hours
10. Jaundice of palms or soles
11. Newborns less than 35 weeks of gestation
12. Newborns birthweight less than 2000 grams

If the newborn temperature is between 95.9°F and 97.5°F kangaroo mother care at home will be advised ^21^. CHWs will also assess for the following maternal danger signs and refer the mother to an appropriate medical facility if any danger sign is present ^21^.

1. Any increase in vaginal bleeding
2. Any history of convulsion after delivery
3. Fast or difficulty breathing
4. Chest pain
5. Fever
6. Too weak to get out of bed
7. Severe headache

CHWs will make a lactation assessment of the feeding of the newborn and offer the mother tips to improve breastfeeding.

**Intervention 3: Home management of neonatal hyperbilirubinemia by LED phototherapy**

The following are the inclusion criteria for newborn home phototherapy. At least one must be present:

a) TcB above the AAP phototherapy threshold adjusting for gestational age and presence of rhesus or blood type incompatibility ^14^.
b) TcB rising ≥ 0.2 mg/dL/hour
The following are the exclusion criteria for newborn home phototherapy:
a) Birthweight <2000g
b) Gestational age < 35 weeks
c) Newborn danger signs present
d) Maternal danger signs present
e) TcB ≥ 15 mg/dL

All newborns will be treated with phototherapy for a 48-hour period. Newborns will be recommended to be placed under the phototherapy lights unless they are feeding, which we estimate to be 30 minutes every three hours with a six-hour phototherapy break overnight. The total treatment duration will be approximately 36 hours of phototherapy, 18 hours per day. After diagnosing the need for home phototherapy, the CHW will initiate phototherapy within four hours. 

 Newborns will wear eye coverings during phototherapy treatment that the CHW will train parents to use. CHWs will instruct parents to turn the device off at midnight and on again at 6 am. If there are issues with the use of the device, parents can call the CHW or research physician with questions. Parents will be instructed when to seek emergency care. The CHW will survey mothers during phototherapy to explore potential issues with treatment.

The phototherapy device will be checked before each use in the home using the manufacturer recommended irradiance meter (Lightmeter V7.0) to ensure that the irradiance is at recommended levels of at least 50 µW/cm ^2^/nm. The phototherapy unit will be covered by a white blanket to maintain the temperature and to enhance phototherapy effectiveness. We will monitor the temperature inside the phototherapy unit with a target of 25 °C under the blanket inside the unit. A portable room heater will be available to maintain the room temperature during phototherapy treatment.

During home phototherapy treatment, newborns will be evaluated by a CHW daily. If any maternal or newborn danger signs are present, home phototherapy will be stopped, and the newborn or mother will be referred to the nearest appropriate health facility. CHWs will answer any questions parents have about the treatment. CHWs will be able to consult the research physician by phone if there are concerns or questions.

***Monitoring after home phototherapy***

After completion of home phototherapy, CHWs will visit the household approximately one day and measure the TcB on two consecutive days and assess for danger signs. We will consider any newborn that has a TcB <15 mg/dL for two consecutive days after treatment and rising <0.2mg/dL/hour to have resolved hyperbilirubinemia. If the TcB ≥ 15 mg/dL or rising ≥0.2mg/dL/hour on the second follow up visit, we will refer the newborn to the hospital for evaluation. If the newborn requires subsequent hospital admission after initial qualification for phototherapy, we will define that as unresolved hyperbilirubinemia.

Transcutaneous bilimeters are not as reliable to measure the progress of phototherapy during treatment, as TcB values are less than the serum value while phototherapy is occurring. After 16–24 hours after phototherapy treatment is completed, their reliability is similar to before phototherapy ^22, 23^. We will perform TcB measurements on consecutive days beginning 24 hours after phototherapy to prove that the transcutaneous bilirubin remains below the treatment threshold and not rising rapidly.

If the family refuses treatment at home, the newborn will be referred to the hospital for treatment. If they refuse home and hospital treatment, CHWs will continue to follow up with the newborn and make three consecutive daily visits in each household and refer the newborn to the hospital if indicated.

***Mobile health (mHealth) data collection and decision support***

**Figure 4.**
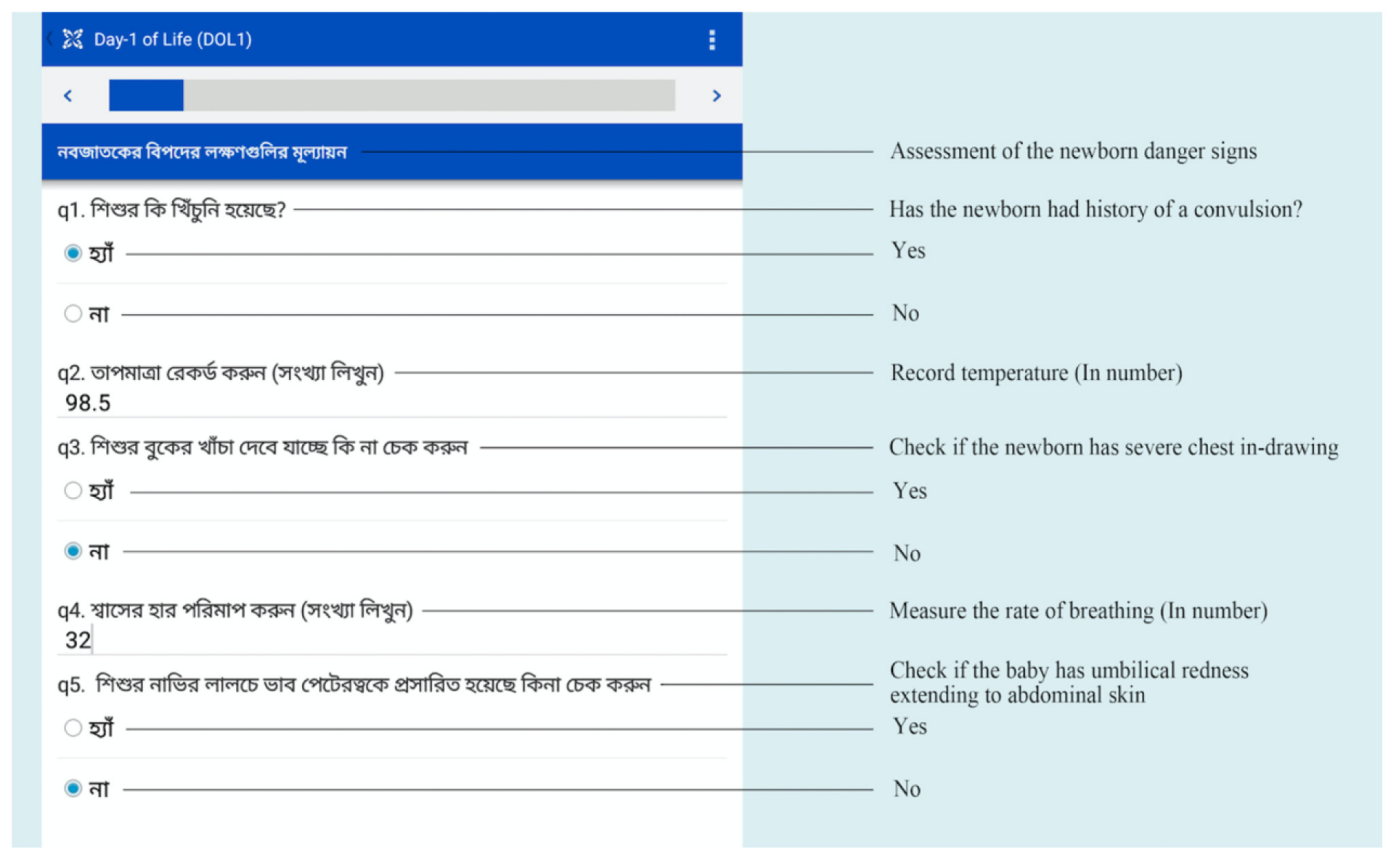
Mobile health CommCare platform with newborn danger sign assessment (Source: icddr,b).

CHWs will use a program that we developed on CommCare (Dimagi, Cambridge, USA) that is uploaded on electronic handheld tablets to guide CHWs through each task during each encounter with participants ( Figure 4). The program will identify which study arm the participant is in and follow the protocols for that arm. The program will guide CHWs on data collection including newborn age in hours calculation, newborn and mother danger sign assessment as well as TcB measurement and management. The application will calculate an estimated delivery date based on the first day of the last menstrual period collected at the baseline survey and add 280 days. The gestational age will be calculated by subtracting the birth date from the first day of the last menstrual period. The age in hours of TcB measurement will be based on the mother informing the CHW the time of birth and by reviewing any delivery records. CommCare will then calculate the age in hours based on the current time and subtracting the time of birth. The age in hours, TcB, gestational age, blood group or rhesus incompatibility between the mother and the newborn will determine the phototherapy threshold for each newborn based on AAP guidelines and hours of life for the newborn ^14^. The data on the blood and rhesus type will be entered and stored in CommCare at the time of collection. The rate of rise of TcB will be calculated by CommCare by subtracting the TcB measured the previous day from the current day’s TcB and dividing by the hours that elapsed between the two measurements.

On completion of newborn danger sign assessment, transcutaneous bilirubin measurement, and maternal danger sign assessment, CommCare will help the CHW decide if the newborn needs to be referred to the hospital for care or can be treated at home with phototherapy for neonatal hyperbilirubinemia. CHWs will be encouraged to consult the research physician by phone or in person if there are concerns or questions.

***Laboratory evaluation of all newborns referred for danger signs or TcB ≥15***

We will obtain the following laboratory studies for any newborn referred for hospital evaluation for danger signs or TcB≥ 15 mg/dL: blood glucose, total serum bilirubin and blood culture. Blood glucose will be tested using a rapid field test kit. Hypoglycemia will be defined as serum glucose < 50 mg/dL. Serum bilirubin will be tested in Sakhipur Health Complex using a Garnier G-3000 semi-auto analyzer and end point, kinetics, fixed-time, 2-point kinetic, absorbance coagulation method.

Blood cultures will be collected by trained medical technologist from icddr,b with assistance of trained staff nurses of Sakhipur Health Complex. At least 1 mL of blood will be collected and the specimen will be transferred to icddr,b laboratory within 12 hours of collection. If *Candida* or multiple bacterial species were identified, the case will be discussed with the research physician and blood cultures will be repeated as indicated to verify the growth as an infection versus the result of contamination. Coagulase-negative *Staphylococcus*, *Diptheroids*, and *Bacillus* species are always considered to be contaminants. Culture reports of growth or no growth will be provided to the clinical care team within 24 hours. Blood culture measurement will help validate CHW danger sign assessments and to determine if infection may have been a cause for neonatal hyperbilirubinemia.

There will be a research physician who will coordinate with hospital and district level managers for any referral issues. Both secondary and tertiary level facility service providers will receive orientation on the study protocol and referral processes. Any newborn who requires exchange transfusion will be referred to Dhaka Shishu Hospital in Dhaka, Bangladesh.

During trial implementation, a field monitoring team consisting of a field research officer, a research physician, and a field coordination manager will regularly visit the field to monitor the intervention delivery. The primary responsibility of this team is to monitor if the intervention has been delivered following guidelines, identify, and solve problems regarding field implementation and report to the investigators.

***Equipment***

Transcutaneous bilirubin will be measured by Draeger JM-105. Draeger JM-105 is a non-invasive transcutaneous bilirubinometer which has demonstrated high accuracy when compared to total serum bilirubin ^24^. Phototherapy treatment will be provided by a double surface portable LED phototherapy device with battery backup, Firefly ( Figure 3). Peak wavelength 455 to 470 nm. Lamp duration 44,000 hours to 30% degradation. Top light: irradiance 34.8 µW/cm ^2^/nm, surface area: 53 cm x 25 cm, irradiance uniformity ratio 0.51 (IEC Compliant > 0.4). Bottom light: irradiance 50.4 µW/cm ^2^/nm, surface area 50 cm x 20 cm irradiance uniformity 0.72. We will measure the irradiance of the device before starting phototherapy. We will also provide a portable room heater to maintain the room temperature at home during phototherapy. The LED phototherapy device has an internal battery backup so it can run in case of power outages.

### Comparison arm (Existing postnatal care system)

In the comparison arm, newborns will receive treatment for neonatal hyperbilirubinemia based on the current postnatal care system in rural Bangladesh. Approximately 7% of newborns born at home have a postnatal visit within 48 hours and are unlikely to be evaluated for neonatal hyperbilirubinemia in a time frame that would result in timely treatment. Infants born vaginally in a hospital delivery often spend 4–8 hours in the hospital prior to discharge. Infants born by vaginal delivery in the hospital or by caesrean section typically have a postnatal care visit by two days of age. If the mothers have a concern about the health of their newborn including concern for jaundice, they may bring the child to the healthcare system leading to physician assessment, and recommendation for testing.

### Evaluation plan

***Baseline survey***

A group of field research assistants will conduct a baseline survey of all mothers at the time of enrollment. The survey will assess each mother’s previous medical history, family history of treatment for hyperbilirubinemia, family history of disability from hyperbilirubinemia, family history of hearing loss. The survey will also collect information on parental socioeconomic factors, knowledge of breastfeeding and neonatal hyperbilirubinemia, delivery plans, and plans for emergency neonatal and maternal care. Figure 1 illustrates the various steps for each arm in the study.

***Birth survey***

We will establish a direct contact system to inform the research team about a study participant’s delivery. The mother and the head of the household will be given the emergency contact number of the research team. They will be instructed to inform the research team once the mother is in labor. The survey will occur approximately one day after discharge of the hospital or by two days of age for home births in intervention households and by seven days of age in control households. The assigned field worker will go to the home and survey the family on the circumstances of the birth, inquire on how long after birth breastfeeding was initiated, measure the birth weight, and calculate the gestational age based on last menstrual period and birth date with the assistance of an electronic tablet.

***Endline survey***

We will carry out a survey in each study arm at four weeks of age to evaluate the health status of the newborns. We will survey for newborn hospitalizations, maternal hospitalizations, presence of neonatal hyperbilirubinemia, screening for neonatal hyperbilirubinemia with blood or TcB measurement, phototherapy treatment for hyperbilirubinemia, neonatal sepsis, exclusive breastfeeding percentage, and maternal depression. The endline survey data will be used to evaluate if the newborn received phototherapy treatment for hyperbilirubinemia.

**Primary outcome**

The primary outcome will be the proportion of neonates that receive hyperbilirubinemia treatment, either at home or in a facility in each study arm.

At the end of this intervention period, we will compare the primary outcomes using a t-test analysis while adjusting for cluster study design between the intervention and control newborns. The significance level will be p <0.05. The quantitative analysis will be conducted by the original assigned groups in an intention-to-treat analysis. Investigators will have no access to outcome data until field activities are complete. CONSORT guidelines will be followed to conduct the analysis ^25^. This analysis will be conducted by the scientific team using a variety of software including R Project for Statistical Computing (RRID:SCR_001905) and STATA RRID:SCR_012763). We are not planning an interim analysis.

**Secondary outcomes**

Secondary outcomes include the proportion of mothers who initiated breastfeeding within one hour of childbirth in each study arm, the percentage of mothers exclusively breastfeeding at four weeks in each study arm, the proportion of neonates born vaginally that are screened for neonatal hyperbilirubinemia by 48 hours of life in each study arm, and any neonatal deaths during that period in each study arm. At the end of this intervention period, we will compare these secondary outcomes using a t-test analysis while adjusting for cluster study design between the intervention and control newborns. The significance level will be p <0.05. The quantitative analysis will be conducted by the original assigned groups in an intention-to-treat analysis.

We will also evaluate for safety measures in Table 2. This includes the number of referrals for danger signs after starting home phototherapy, the need for exchange transfusion after starting home phototherapy, the development of extreme hyperbilirubinemia (total serum bilirubin ≥25 mg/dL) after starting home phototherapy and severe hyperbilirubinemia (total serum bilirubin ≥20 mg/dL) after starting home phototherapy. We will measure the number of newborns with sepsis (positive blood culture) diagnosed after starting home phototherapy, and the number of newborns diagnosed with hypoglycemia (serum blood glucose ≤ 50mg/dL) after starting home phototherapy.

**Table 2.** Safety outcomes assessed after home phototherapy was started.

% newborns with resolved hyperbilirubinemia after starting home phototherapy
% newborns referred to the hospital for treatment for phototherapy at the first visit after completing home phototherapy
% newborns referred to the hospital for treatment for phototherapy at the second visit after completing home phototherapy
% newborns with total bilirubin ≥ 20mg/dL
% newborns with total bilirubin ≥ 25mg/dL
% newborns receiving exchange transfusion
% newborns exclusive breastfeeding
% newborns with any formula feeding
% completed 36 hours of phototherapy
% newborns receiving intravenous fluids
% newborns with hypothermia (temperature <35.5°C)
% of newborns whose mothers refused treatment after starting home phototherapy
Number of newborn deaths
Number of newborns with acute bilirubin encephalopathy
% of newborns with sepsis diagnosed
Number of newborns with positive blood culture
% of newborns diagnosed with hypoglycemia

### Missing data

CHWs will communicate with the participants throughout the study over the phone to monitor their health condition. This will build a sense of trust and reliability between study participants and CHWs. The research team will also arrange community meetings to gain overall community and community leader support on neonatal hyperbilirubinemia screening and management. This outreach will be integral to encouraging protocol adherence. However, in the case of protocol non-adherence we will use an intention to treat analysis to assess the main study outcome. For any other missing data, we will document the cause of missing data. For analysis, we will not count the missing data and will set the denominator excluding the missing data

### Data and safety monitoring board (DSMB) and safety review plan

The protocol and informed consent will be reviewed by Stanford University and icddr,b committees on human research and ethical review committees.

An independent DSMB will be assembled in Bangladesh to monitor adverse events and to advise investigators with five respective members. The board will be composed of two neonatologists, two epidemiologists, and one demographer. Stanford University institutional review board will also review the protocol prior to beginning the study.

We will collect data on treatment progress, side effects of treatment, adverse events during the study. Study team listed a number of indicators that will be monitored to identify any serious adverse events and will be reported to DSMB. The indicators to be monitored and reported are listed below, as specified by the research team:

i) Number of newborns referred to the hospital due to danger signs after starting phototherapy at home
ii) Number of newborns died from to neonatal hyperbilirubinemia at home or hospital after starting phototherapy
iii)  
iv) Number of newborns that developed severe hyperbilirubinemia (total serum bilirubin ≥ 20 mg/dL) after starting home phototherapy
v) Number of newborns that developed extreme hyperbilirubinemia (total serum bilirubin >=25 mg/dL) after starting home phototherapy
vi) Number of newborns needing exchange transfusion after starting home phototherapy
vii) Number of newborns with acute bilirubin encephalopathy after starting home phototherapy
viii) Number of newborns with sepsis diagnosed after starting home phototherapy
ix) Number of newborns diagnosed with hypoglycemia after starting home phototherapy.
x) Number of newborns with hypothermia after starting home phototherapy

Any cases of acute bilirubin encephalopathy, exchange transfusion, or death during the trial will be considered a serious adverse event and reported to DSMB. The investigators will report serious adverse events to the DSMB within 24 hours of the event. For any serious adverse event a follow-up report will be submitted withing 48 hours and a final report will be submitted within 72 hours of first report submission. The DSMB will be responsible for evaluating the event and decide if the principal investigator’s report is satisfactory or not. . If the event is not a serious adverse event but determined by the principal investigator to be reportable, an initial report will be submitted to the DSMB as soon as possible, but no later than seven days after the investigators learns of the event. The DSMB will disseminate the outcome of the review to principal investigator of the study.

Side effects from phototherapy treatment are rare and the AAP regards the treatment as safe ^14^. A rare complication of phototherapy, bronze baby syndrome, occurs in some infants with cholestatic jaundice when treated with phototherapy ^26^. With exposure to phototherapy, infants develop a dark, gray-brown discoloration of skin, urine, and serum. For newborns with a bilirubin above the phototherapy threshold it is recommended to treat infants with cholestatic jaundice with phototherapy. The incidence of cholestatic jaundice in newborns is 1 in 2500 ^27^. Not all newborns with cholestatic jaundice need phototherapy and the long terms effects of bronze baby syndrome are not thought to be deleterious and resolve over time. CHWs will screen and monitor to determine the possibility of the development of this complication.

Another possibility is the development of purpura or bullae in infants with cholestatic jaundice or congenital erythropoietic porphyria ^28^. There are approximately 200 cases of congenital erythropoietic porphyria reported. Because the photosensitivity and blistering can be severe in infants with porphyria, infants who have this diagnosis or a positive family history for this disorder have an absolute contraindication for phototherapy. CHWs will screen and monitor neonates to determine the possibility of the development of this complication.

### Qualitative analysis

We will use focus group discussions (FGD) and in-depth interviews (IDI) to assess barriers and facilitators of home treatment and hospital treatment amongst the implementers and the participants. Discussions will assess for challenges with using the phototherapy device and explore mothers’ concerns with the treatment and whether they felt they could ask for help in using the device. We will explore if mothers felt like using the device made breastfeeding more difficult. Facilitators will query if CHWs provided adequate explanations for why treatment was necessary and if they educated the mother sufficiently on how to do the treatment.

For mothers whose newborns were referred to the hospital for treatment, we will assess if there was difficulty breastfeeding due to treatment, if there was difficulty traveling to the hospital, and if the hospital provided adequate resources for treatment. We will discuss barriers and facilitators of a successful referral to the hospital. Facilitators will query if CHWs provided adequate explanations for why referral was needed.

Facilitators will explore mothers’ comfort with CHWs and their trust in their recommendations. We will explore if diagnosis of hyperbilirubinemia needing treatment with the transcutaneous bilimeter was more likely to result in successful referral in comparison to diagnosis with danger signs by CHW physical exam.

***Data collection plan for qualitative analysis***

The qualitative data will be collected by qualitative researchers, and they will be trained on the proposed guideline before going to the field. The collection plan is outlined in Table 3. All the IDIs and FGDs will be recorded using audio recorders. The research team will take field notes of informal discussions, observations on the tone and attitudes of the respondents during data collection.

**Table 3.** Selection criteria for qualitative analysis of hyperbilirubinemia management.

	Participant	N	Selection Criteria
IDI	CHWs	10	All CHWs
	Mothers	60	Convenience, 20 whose newborns were recommended for home phototherapy, 20 in the hospital referral arm whose newborns were otherwise healthy and referred to the hospital for treatment, 20 whose newborn was referred for danger signs or TcB ≥15
	Hospital health worker	10	Hospital physicians, nurses, and hospital administrators will selected by convenience
	CHW Supervisors	3	All supervisors will be interviewed
FGD	Parents, grandparents	80	Geographic area, Mothers not participating in in-depth interviews

IDI – in-depth interviews, FGD – focus group discussions

Audio recorded data of IDIs and FGDs will be transcribed into Bengali and then translated to English. Some portions of the interviews that contain local terms and expressions will be highlighted to understand the tone of the interviewees. Code lists for each of the tool; *i.e.,* FGD, IDI will be prepared separately. All the data will be coded accordingly using ATLAS.ti version no. 5.2. Then coded data will be summarized according to the study objectives and relevant themes. All data will be analyzed considering the content and context analysis, followed by comparison and triangulation. It is common phenomenon to have different findings when applying different tools for the same issue with the same unit of analysis. If this occurs, we will conduct additional exploration.

One assistant program manager will regularly check the data and will identify any inconsistency, missing data and quality of the data and will report to the investigators.

### Ethics and dissemination

This protocol ( Clinical Trial (CT) registration ID # NCT03933423) and informed consent was reviewed and approved by icddr,b and Stanford University committees on human research and ethical review boards (PR-19004, 52625). Any modifications to the protocol will be communicated to the institutional review boards to be approved for ethical clearance. Trained field research assistants from icddr,b will obtain written informed consent from participants. The purpose of the study, methods and procedure, risk and benefits, confidentiality and future use of information will be explained to the participants. For the collection and use of biological specimen, additional informed consent will be taken from the participants.

Trial data will be collected in the CommCare database, which is a secure, web-based application designed to support data capture for research studies. All pregnant women and newborns will have an anonymous study identification number. Any documents linking patient identifiers to the anonymous study identification number will be stored in CommCare.

Both icddr,b and Stanford research teams will have access to final trial data and there are no contractual agreements that limit access to investigators. Only aggregate analyses without patient identifiers will be published. The study data will be stored by the principal and co-investigators during study period and will be stored in the icddr,b data repository under icddr,b data repository committee at the end of the study period. Data from the icddr,b Data Repository (icddr,b Datasets) will be provided upon request for purposes of secondary data analyses upon approval of a Data Licensing Application & Agreement. The Spirit 2013 Checklist and CONSORT 2010 checklist were uploaded to Zenodo ^25^. To maintain participants’ anonymity and confidentiality, the data set generated during the study will not be publicly available but are available from the principal investigator on reasonable request. We plan to disseminate the results of this trial in peer-reviewed journals and international conferences. Our target audience are those involved in the of newborn health management in low-resource settings as well as those who develop and advise on policy, especially the Ministry of Health and family Welfare, Bangladesh.

We will periodically share the progress and challenges of the study to keep the stakeholders updated and seek their support as and when needed. At the end of intervention, we will convene a national level stakeholders’ meeting to disseminate study findings with government and non-government organizations. This convening will also be an opportunity to influence implementers based on the result generated by the project. We will develop an agenda for the convening meeting that addresses key themes that emerge from household-level interventions.

### Study status

The study has completed data collection and the results are being analyzed. No changes were made to the protocol after the trial commenced.

## Discussion

In this study, we will evaluate the impact of CHW-led home screening and household phototherapy treatment for neonatal hyperbilirubinemia on access to phototherapy treatment in a rural community in Bangladesh where timely postnatal care is limited. The treatment arm will employ improved household screening with a point of care transcutaneous bilirubin (TcB) measurement, a mHealth platform to extend the capabilities of CHWs, and a phototherapy device that can be used to treat newborns at home. This study leverages relationships with Bangladeshi communities, the government, and health providers.

The results of the study will be clinically relevant by developing evidence to show how to successfully expand access to neonatal hyperbilirubinemia care to the most vulnerable newborns in LMICs. It will provide evidence on how to safely extend the capabilities of CHWs through mHealth with a validated treatment algorithm to provide quality postnatal care and curative neonatal hyperbilirubinemia care. LMICs are expanding access to postnatal care by using CHWs, and our work will give CHWs a curative treatment option in the immediate postnatal period. Caring for newborns at home instead of in the hospital offers potential benefits including improved access and acceptability which may lead to improved health outcomes as well as decreased risk of obtaining hospital acquired infections, which could drive widespread adoption and reduce morbidity and mortality from neonatal hyperbilirubinemia care amongst those that are most vulnerable. Similar projects in other LMICs can be pursued to evaluate their effectiveness and dramatically extend healthcare access to the most vulnerable newborns.

Prior work has shown that CHWs can accurately detect severe illness in newborns after birth and prevent mortality through postnatal visits screening for neonatal danger signs *via* physical assessment ^19^. CHW home visits in the first days after birth can detect in rural Bangladesh reduce newborn mortality by 34% ^9, 29, 30^. The performance of CHW physical exams to detect clinical danger signs in newborns was validated and the exams had 81% sensitivity and 96% specificity to detect clinical danger signs ^19^. This research has influenced the WHO guidelines to integrate seven newborn danger signs as a part of the Integrated Management of Childhood Illness guidelines and we used these danger signs as a part of our algorithm to manage neonatal hyperbilirubinemia ^7, 17^. We will use this validated screening method to identify newborns without danger signs and are otherwise healthy and can safely be treated with home phototherapy. We also screen for maternal danger signs to assess the ability of the mother to successfully provide care at home ^18^.

The home screening and treatment protocol was developed by applying evidence on neonatal hyperbilirubinemia screening to reduce the incidence of severe hyperbilirubinemia in rural Bangladesh. In South Africa, a study estimated that for newborns born in the hospital, universal newborn TcB screening prior to discharge from the hospital reduced the risk of severe hyperbilirubinemia by 73% in comparison to physical exam screening ^8^. Newborns were screened only one time with a TcB at the time of discharge and vaginal births were discharged after six hours and caesarean section births were discharged after 48 hours ^8^. With more TcB screenings and beginning at 24–48 hours of life we believe this program will ultimately reduce hyperbilirubinemia-related disability and death. Universal hospital-based bilirubin screening in the US has reduced the incidence of hyperbilirubinemia above the exchange transfusion threshold ^11^.

The home screening and treatment arm will rely on mobile health to extend the capabilities of CHWs to manage hyperbilirubinemia at home. Mobile health applications have been used by CHWs in LMICs to improve newborn care ^31^. mHealth has been shown to improve the accuracy of CHW newborn clinical assessments, improve the completion of assessments, their speed, and the adherence to clinical management guidelines with high satisfaction ^31^. In particular, mHealth has been used by CHWs to improve the classification and management of hypothermia and infant feeding problems ^31^. We will use mHealth to improve the management of newborns after birth and extend the capabilities of CHWs to improve management of neonatal hyperbilirubinemia.

The home screening and treatment arm leverages evidence-based strategies to reduce the risk of rebound hyperbilirubinemia after treatment and increase the likelihood of resolution of hyperbilirubinemia with CHW-led home treatment. Chang *et al.* found that diagnosing the need for phototherapy early and treating early reduces the risk of rebound hyperbilirubinemia by about 50% per day ^32^. In addition stopping treatment after longer phototherapy durations when the difference between the bilirubin on stopping treatment and the bilirubin treatment threshold is larger reduces the risk of rebound hyperbilirubinemia by about 50% for each mg/dL decrease in bilirubin ^32^. We begin TcB screening early, by day of life two and screening daily for 3 days. We will diagnose and treat cases of hyperbilirubinemia early reducing the risk of rebound hyperbilirubinemia. CHWs will treat with high intensity phototherapy (50 μW/cm ^2^/nm) for 36-hour total treatment duration ( Figure 2). The 36-hour treatment duration will decrease the bilirubin below the treatment threshold and reduce the risk of rebound hyperbilirubinemia and hospital referral after phototherapy. Treatment with high intensity phototherapy with irradiances >30 μW/cm ^2^/nm cause a faster and larger declines in bilirubin than phototherapy of lower intensity and cause a 50% reduction in the first 24 hours of treatment ^14^. In a past study of a population of newborns that needed phototherapy, 82% completed treatment within a single hospitalization with a typical duration phototherapy of 22–27h using 22–25 μW/cm ^2^/nm phototherapy ^33^. LED phototherapy has minimal side effects and uses light of blue wavelength (455–470nm) to help lower the concentration of bilirubin in the body ^14^. We propose a 6-hour break overnight so that the mother does not have to monitor at night. Intermittent phototherapy is successful in reducing hyperbilirubinemia ^14^.

Accessing neonatal hospital-based care can be a barrier for families in LMICs and home-based care has been used to increase the percentage of newborns receiving the recommended treatment. In a prior study, only one-third of newborns in rural Bangladesh that were referred to the hospital from home for physical exam findings concerning for sepsis completed the referral ^9^. The costs of traveling to the hospital and obtaining care were found to be a major barrier ^9, 10^. However, families were willing to have their newborns treated at home. Approximately 65% of parents who refused referral for neonatal sepsis evaluation in hospitals, consented to home care by CHWs with intramuscular injection with antibiotics, increasing access to care and reducing neonatal mortality by 34% ^9^. Because we are increasing access to hyperbilirubinemia treatment by treating newborns at home, we believe that the home screening and treatment arm will treat more newborns than the existing system in Bangladesh and has the potential to prevent morbidity and mortality from neonatal hyperbilirubinemia.

## Data Availability

Zenodo: Community health worker led household screening and management of neonatal hyperbilirubinemia in rural Bangladesh: a cluster randomized control trial protocol Spirit 2013 Checklist, Consort 2010 Checklist, and Consent forms https://doi.org/10.5281/zenodo.7488226 ^25^. This project contains the following extended data: Consent Form for participants enrollment.docx Zenodo: Community health worker led household screening and management of neonatal hyperbilirubinemia in rural Bangladesh: a cluster randomized control trial protocol Spirit 2013 Checklist, Consort 2010 Checklist, and Consent forms https://doi.org/10.5281/zenodo.7488226 ^25^. Consort 2010 CHecklist12_9.doc Spirit checklist_Homephoto trial protocol12_27.doc Data are available under the terms of the Creative Commons Attribution 4.0 International license (CC-BY 4.0).
