# Supplementary material for: Community health worker-led household screening and management of neonatal hyperbilirubinemia in rural Bangladesh: a cluster randomized control trial protocol
Source: Gates Open Res. 2024 Jan 5;7:58. Originally published 2023 Apr 21. [Version 2] doi: 10.12688/gatesopenres.14033.2 (PMC11249711; doi:10.12688/gatesopenres.14033.2)
Supplement: Peer review history

# Reviewer report: Reviewer response for version 2

**DOI:** 10.21956/gatesopenres.16516.r36817

**Published:** 2024-07-15

**Authors:** Jean-Baptiste Le Pichon

**Affiliations:** 1 Children’s Mercy Hospital, Kansas City, MO, United States Virgin Islands

In this paper, Foote et al. develop an intervention protocol to identify and treat newborn hyperbilirubinemia in a province of rural Bangladesh. The proposed intervention is a cluster randomized trial of 530 families randomly assigned to routine care or home visits pre- and postpartum by clinical health workers (CHW).

**Strengths:** This study addresses a major cause of mortality and morbidity in LMICs. The intervention proposed is cost-efficient and feasible in an LMIC. The results are actionable and, if successful, will immediately benefit the affected populations. The proposal is well-designed and relies on an existing solid medical and social infrastructure (a well-organized, already-existing system of registration and follow-up of pregnant women in the Sakhipur sub-district of Bangladesh).

 The authors propose three specific aims targeted to measure the effectiveness of:

1. Parental education in the prevention of disease
2. Screening in detecting treatable hyperbilirubinemia
3. Home treatment for hyperbilirubinemia.

The specific aims are clear and independent of each other. However, the SA's order is somewhat counterintuitive. I suggest rearranging them to follow the more logical prevention, intervention, and treatment order (as outlined above).

**Areas of possible improvement:** The authors anticipate a 5% loss to follow-up. This is an unusually low rate, even in high-income countries. In LMICs this is expected to be much higher. For example, in one study of infants conducted in Chandigarh India, more than ½ of the cohort was lost to follow-up by the end of one year (Mathew et al, 2021)(Ref-1). While it will greatly help in the present study that it is on a much shorter time scale and the CHW will be visiting the homes, a loss of follow-up of 5% still seems unrealistically optimistic.

 It is surprising that there is no discussion of G6PD. G6PD is a major risk for bilirubin encephalopathy (Olusanya et al, 2014)(Ref-2). There are several point of care G6PD tests that could be easily added to this protocol at a minor cost.

 The proximity cluster design is ingenious and well thought out, but it introduces a small risk of sample bias (for example, the cluster proximity could result in women influencing each other’s behaviors or low heterozygosity within the cluster that could result in an unusually high rate of G6PD within a cluster). It would be good if the authors addressed this issue as a potential limitation.

 It would help to have a better description of how the authors plan on blinding the outcome assessors. As it stands, the only method for binding the outcome assessors is to have “minimal contact” with the CHWs. How will the assessors be blinded to knowledge of the intervention? Will they have any contact with the mothers? Will they see only medical records? If so, what medical records will the assessors see?

 The protocol for home interventions is solid and well thought through. The authors readily acknowledge the limitations of TcB measurements and address these limitations effectively. However, it is surprising that no mention is made anywhere of the Bilirubin Induced Neurologic Dysfunction (BIND) scale (Radmacher, et al., 2015)(Ref-3). The modified BIND scale is a proven and effective way to assess the risk for acute bilirubin encephalopathy in newborns . It was also shown recently to be highly sensitive in predicting of kernicterus (Gelineau-Morel et al.,2023)(ref-3).

**Minor issues:**

1. In the abstract, the authors state that “Newborns without danger signs but with a TcB above the treatment threshold, but >15 mg/dL will be treated with light-emitting diode (LED) phototherapy at home”. I believe they meant “Newborns without danger signs but with a TcB above the treatment threshold, **but <15 mg/dL** will be treated with light-emitting diode (LED) phototherapy at home.

The abbreviation for clinical health worker (CHW) is never defined.

Is the study design appropriate for the research question?

Yes

Is the rationale for, and objectives of, the study clearly described?

Yes

Are sufficient details of the methods provided to allow replication by others?

Yes

Are the datasets clearly presented in a useable and accessible format?

Yes

Reviewer Expertise:

Hyperbilirubinemia, bilirubin encephalopathy, kernicterus spectrum disorder, epilepsy, neurogenomics.

I confirm that I have read this submission and believe that I have an appropriate level of expertise to confirm that it is of an acceptable scientific standard.

## References

[1] : Analysis of reasons for loss to follow up in a prospective study in Chandigarh, India and impact from telecom changes. *BMC Res Notes* .2021;14(1) : 10.1186/s13104-021-05837-9 419 10.1186/s13104-021-05837-9 34794507 PMC8600768

[2] : Addressing the burden of neonatal hyperbilirubinaemia in countries with significant glucose-6-phosphate dehydrogenase deficiency. *Acta Paediatr* .2014;103(11) : 10.1111/apa.12735 1102-9 10.1111/apa.12735 24990658

[3] : A modified Bilirubin-induced neurologic dysfunction (BIND-M) algorithm is useful in evaluating severity of jaundice in a resource-limited setting. *BMC Pediatr* .2015;15: 10.1186/s12887-015-0355-2 28 10.1186/s12887-015-0355-2 25884571 PMC4389967

[4] : Predictive and diagnostic measures for kernicterus spectrum disorder: a prospective cohort study. *Pediatr Res* .2024;95(1) : 10.1038/s41390-023-02810-z 285-292 10.1038/s41390-023-02810-z 37689774 PMC10842628

# Reviewer report: Reviewer response for version 2

**DOI:** 10.21956/gatesopenres.16516.r35758

**Published:** 2024-05-31

**Authors:** Tina M. Slusher, Zubaida Farouk

**Affiliations:** 1 Department of Pediatrics, Global Health Program, Critical Care Division, University of Minnesota, Minneapolis, MN, USA; 2 Bayero University Kano, Kano, Nigeria

The revisions that the authors have made have addressed many of my/our concerns but there are still a few that need to be addressed.  They have re-ordered their aims which was an essential correction needed in this arm, further clarified community health worker training and included the bilirubin graph and access to cell phone.  

 The authors still have not addressed the problem of G6PD deficiency and make no mention of screening for G6PD deficiency.   If they cannot screen for G6PD deficiency that should be stated and authors should mention at a minimum that mothers will be taught to avoid triggers of hemolysis.

 Again it would be helpful to know the outcomes of the control group that have EBT, ABE, or jaundice-related death.  This data will be very important when determining the success of the home PT and should be relatively easy to obtain.   Additionally it would be helpful to state how the diagnosis of ABE will be made and BIND score encouraged for any neonate referred to the hospital for treatment of jaundice.

 Please clarify that the distance from the neonate to the PT will be fixed and not adjustable by the mother/family.

 Thank you for this very important work.  If shown to be safe and effective it will likely be an important tool in preventing severe neonatal hyperbilirubinemia and the tragic sequelae including ABE and KSD.

Is the study design appropriate for the research question?

Yes

Is the rationale for, and objectives of, the study clearly described?

Yes

Are sufficient details of the methods provided to allow replication by others?

Partly

Are the datasets clearly presented in a useable and accessible format?

Not applicable

Reviewer Expertise:

NA

We confirm that we have read this submission and believe that we have an appropriate level of expertise to confirm that it is of an acceptable scientific standard, however we have significant reservations, as outlined above.

# Reviewer report: Reviewer response for version 1

**DOI:** 10.21956/gatesopenres.15331.r34000

**Published:** 2023-08-03

**Authors:** Christian V Hulzebos

**Affiliations:** 1 Beatrix Children's Hospital, University Medical Center Groningen, Groningen, The Netherlands

In this study protocol entitled “Community health worker-led household screening and management of neonatal hyperbilirubinemia in rural Bangladesh” Foot and colleagues scrutinously describe their research proposal to improve management of neonatal hyperbilirubinemia (NHB) in a low-resource country.

 The authors aim to prevent NHB by antenatal education on the importance of breastfeeding and inform mothers how to seek help when their offspring is sick, to prevent NHB by transcutaneous bilirubin (TcB) screening at home, and to start early phototherapy (PT). PT is started at home whenever TcB < 15 mg/dL (257 µmol/L) is higher than the treatment threshold, and in-hospital when TcB ≥ 15 mg/dL or in case of danger signs.

 Considering the important aim on prevention I suggest to adapt the title, and incorporate prevention in the title as well! And please incorporate this in the intervention arm as well. For example in the abstract the intervention arm reads as home screening and treatment, which is in fact not completely true.

 I agree with the other reviewers that this study does have potential to change the future of many infants with NHB. Yet, apart from the scientific issues, economic and financial factors are as well important and need some clarification. An important question that arises is how resources are guaranteed after this study with a clear but relatively small sample size. And what about future costs for regular calibration of TcB and PT devices?

 I have also a question on the effectiveness of home PT to increase treatment rates (as can be read in the abstract). The authors have defined treatment rate as primary outcome. But this is not the same as PT effectiveness, usually defined as reduction of bilirubin below the treatment threshold that is considered appropriate. Especially when treatment is TcB based < 15 mg/dL, and not TSB-based, overtreatment may occur. And this unnecessarily started PT will contribute to the primary outcome. Therefore I suggest to measure TSB also in newborns with TcB above treatment threshold < 15 mg/dL. In this way, resources will be reserved for newborn infants with clinically relevant hyperbilirubinemia, i.e., TSB > treatment threshold.

 I have provided my suggestions and questions in the linked PDF . This PDF also contains the comments from Tina M. Slusher and Zubaida L. Farouk as part of their review of version 1 of this article ^1^.

Is the study design appropriate for the research question?

Partly

Is the rationale for, and objectives of, the study clearly described?

Yes

Are sufficient details of the methods provided to allow replication by others?

Yes

Are the datasets clearly presented in a useable and accessible format?

Not applicable

Reviewer Expertise:

Neonatal hyperbilirubimemia, prevention, screening, treatment, management guidelines.

I confirm that I have read this submission and believe that I have an appropriate level of expertise to confirm that it is of an acceptable scientific standard, however I have significant reservations, as outlined above.

## References

[1] : Peer Review Report For: Community health worker-led household screening and management of neonatal hyperbilirubinemia in rural Bangladesh:a cluster randomized control trial protocol [version 1; peer review: 1 approved with reservations]. *Gates Open Res* .2023;7(58) : 10.21956/gatesopenres.15331.r33065 10.21956/gatesopenres.15331.r33065 Reference source PMC1124971139015822

# Reviewer report: Reviewer response for version 1

**DOI:** 10.21956/gatesopenres.15331.r33065

**Published:** 2023-05-15

**Authors:** Tina M. Slusher, Zubaida Farouk

**Affiliations:** 1 Department of Pediatrics, Global Health Program, Critical Care Division, University of Minnesota, Minneapolis, MN, USA; 2 Bayero University Kano, Kano, Nigeria

This study does have the potential to improve outcome in neonates with significant hyperbilirubinemia in Bangladesh and other LMICs if shown to be safe and effective. Obviously, it should be possible to provide more timely access to phototherapy and to treat neonates who are having difficulty getting to their referral center and to do it less expensively—all very important in LMICs. It is well designed as a cluster randomized trial. The most recent 2022 AAP guidelines do allow for careful and limited home phototherapy but as these authors clearly realize a protocol for home phototherapy in other countries including LMICs needs to be carefully studied for safety and efficacy before widespread implementation. We congratulate these authors for this idea. The study team is strong and quite experienced. We do have questions to which we feel answers will improve the study substantially. Thank you for allowing us to review this protocol.

1. We suggest attaching survey questionnaires and other tools including checklists for the study. These materials would likely fill in many of the gaps we mention below.
2. The focus of the study is timely identification and treatment of hyperbilirubinemia, why should aim 1 be about exclusive breastfeeding? We would suggest reversing the aims or simply including encouraging and supporting breastfeeding as a means to decrease hyperbilirubinemia.
3.  You do not mention training for the mothers on preventative practices like avoiding triggers for hemolysis in G6PD deficient neonates such as avoiding henna and mentholated products or jaundiced preventative measures beyond adequate breastfeeding. G6PD screening should be included in the baseline labs as Bangladesh has a G6PD deficiency rate greater than 5% in males. Without knowing G6PD status determining risk of significant or severe jaundice will be more difficult.
4. We also wonder about neonates with set up for Rhesus or ABO incompatibility? Will these neonates be enrolled in the study and not transferred to the referral center until after 36-48 hours or will they be excluded and referred sooner? We would be okay with starting home phototherapy in these neonates while transport was arranged but would be uncomfortable keeping these neonates on home phototherapy for a prolonged time because of their risk of rapidly developing severe hyperbilirubinemia which might be missed with only daily bilirubin checks.
5. It would be important to know the outcomes of the comparative group i.e., the number in both the treatment arm and control arms that need hospital referral, EBT and have ABE or die from complication of the treatment or jaundice-related death.
6. Are you looking at the proportion of newborns receiving treatment in both groups? Will the comparative or control group ever get home phototherapy? Will both groups be receiving intensive phototherapy?
7.  Agree with need to specifically look for ABE and would suggest either the BIND or modified BIND score in any neonate with possible ABE on exam in both groups. Suggest looking for signs of ABE at the time of starting phototherapy and each time the neonate is examined. You should also assess for ABE at timepoint of referral to the hospital for exchange. We also would strongly suggest surveying/examining for signs of ABE at the endpoint.
8. What is the definition of geographical proximity for the purpose of this study? Suggest minimum distance or some other objective criteria, can individual villages or wards serve as clusters?
9.  What is this written test that the CHW will take? Is an aptitude test? Do CHW have any prior medical training? Are they certified health workers? Please clarify in more detail what assessment the CHW’s will carry out and specify how it will be done.
10.  Please clarify tool that will be used to assess CHWs. What is the qualification of the field staff that will review performance of the CHW, what is the qualification of the training team? What will be used to assess the knowledge, skill, and attitude? Exactly what type of training and specific skill sets of CHW’s varies from country to country therefore needs to be specified.
11. Is the distance between the neonate and the phototherapy lights/lamps fixed in a way that parents cannot adjust the distance otherwise I would be concerned that they might change the distance which of course affect the PT intensity. What does the training on phototherapy device entails? Does it cover how to provide effective phototherapy to an infant, where to place the infant, eye cover, indications for phototherapy, safety issues regarding phototherapy, exposure? Who is responsible for checking the irradiance of the photo units? CHW? Are the CHWs measuring it and if so, will they be trained how to do it accurately? How will the phototherapy be powered at home? Is there constant and availability of electricity at all homes in the study area?
12. Also please explain why you chose to use the AAP guidelines and if they were adapted for use in Bangladesh as strongly suggested by the AAP themselves. 
13. Please state range of values rather than TcB above phototherapy threshold or simply include the graph showing used to determine when phototherapy will be begun. If using the AAP guidelines, then should almost certainly use the more conservative 2004 guidelines not the new 2022 guidelines but it would be much better would be to adapt guidelines specifically for your locale as stated above.
14. Comprehensive guidelines already include jaundice as a danger sign therefore you are already going against these guidelines by keeping the neonate at home therefore you will need to explain this in your protocol. Is visual jaundice on the palm and sole despite TcB a requirement for referral to a hospital in this study? Please clarify.
15. Who will be responsible for checking of the ambient temperature? The parents or CHW?
16. State whether all parents have access to a phone?
17. Please provide a checklist stating the potentials issues that the CHW will be checking.
18. Who is going to do the blood groups CHWs? Are they certified to do heel pricks and run these tests in Bangladesh or is that certification required or is training CHWs to do these test all that is required for them to be able to do them? With training are the CHWs or other study staff able to do these tests in this setting?
19. We are not statisticians, but we do not believe t-test is the appropriate test because t-test is used to compare between means and Chi Square test is used to compare proportions. This would also be true in secondary outcomes.

Please find further comments in the annotated pdf hyperlinked above or at the following link: *https://gatesopenresearch.s3.eu-west-1.amazonaws.com/linked/208927.Annotated_pdf_-_Review_Drs._Tina_Slusher_and_Zubaida_Farouk_for_Home_PT_in_Bangladesh_%28002%29.pdf*

Is the study design appropriate for the research question?

Yes

Is the rationale for, and objectives of, the study clearly described?

Yes

Are sufficient details of the methods provided to allow replication by others?

Partly

Are the datasets clearly presented in a useable and accessible format?

Not applicable

Reviewer Expertise:

NA

We confirm that we have read this submission and believe that we have an appropriate level of expertise to confirm that it is of an acceptable scientific standard, however we have significant reservations, as outlined above.
